# MARCH5 promotes hepatocellular carcinoma progression by inducing p53 ubiquitination degradation

**DOI:** 10.1007/s00432-024-05782-7

**Published:** 2024-06-11

**Authors:** Xin Cai, Jie Gao, Zhiping Yan, Huapeng Zhang, Danfeng Guo, Shuijun Zhang

**Affiliations:** 1 Department of Hepatobiliary and Pancreatic Surgery, The First Affiliated Hospital of Zhengzhou University, Zhengzhou, China; 2 Henan Liver Transplantation Centre, Zhengzhou, China; 3 The Main Construction Unit of National Regional Medical Center for Henan Organ Transplantation, Zhengzhou, China; 4 Henan Research & Development International Joint Laboratory for Organ Transplantation Immunomodulation, Zhengzhou, China; 5 Zhengzhou Key Laboratory for Hepatobiliary & Pancreatic Diseases and Organ Transplantation, Zhengzhou, China

**Keywords:** HCC, Autophagy, MARCH5, p53, Ubiquitination

## Abstract

**Background:**

Human MARCH5 is a mitochondria-localized E3 ubiquitin-protein ligase that is essential for the regulation of mitochondrial dynamics. A large body of evidence suggests that imbalances in mitochondrial dynamics are strongly associated with cancer. However, the expression, biological function and prognostic significance of MARCH5 in hepatocellular carcinoma (HCC) have not been determined.

**Materials and methods:**

The mRNA and protein expression of MARCH5 in HCC cell lines and tumor tissues was assessed by real-time quantitative PCR, Western blot analysis and immunohistochemistry. The clinical prognostic significance of MARCH5 was evaluated in 135 HCC patients. Knockdown or overexpression of MARCH5 in HCC cells was determined by in vitro cell proliferation, migration and invasion assays, and in vivo tumor growth and metastasis assays. In addition, the intrinsic mechanisms by which MARCH5 regulates HCC cell growth and metastasis were explored.

**Results:**

MARCH5 was significantly overexpressed in HCC cells and was closely associated with patients' poor postoperative prognosis. In vivo and in vitro experiments revealed that MARCH5 significantly promoted the increase and invasive and migratory ability of hepatocellular carcinoma cells, which was mainly due to the promotion of autophagy by MARCH5. Mechanistic studies revealed that MARCH5 promoted autophagy through ubiquitination degradation of p53 leading to malignant progression of hepatocellular carcinoma.

**Conclusion:**

Our findings suggest that MARCH5 plays a critical oncogenic role in HCC cells, which provides experimental evidence for the use of MARCH5 as a potential target for HCC therapy.

**Supplementary Information:**

The online version contains supplementary material available at 10.1007/s00432-024-05782-7.

## Introduction

HCC is the main form of primary liver cancer and is one of the most common cancer types, ranking fourth in global tumor-related mortality (Llovet et al. 2021). Different risk factors, such as HBV or HCV infection, cirrhosis, autoimmune hepatitis, nonalcoholic fatty liver disease, and alcoholism, may contribute to the development of HCC (Llovet et al. 2021; Rebouissou and Nault 2020; Villanueva 2019). In addition, many key molecular alterations have been identified during HCC, including TERT, TP53, and CTNNB1, among others (Llovet et al. 2021; Villanueva 2019). HCC cells are extremely proliferative and metastatic, and are susceptible to drug resistance, which contributes to their invasive behavior (Deldar Abad Paskeh et al. 2021). Despite significant advances in clinical treatment, the prognosis for patients with HCC remains poor because of their rapid progression and early relapse (Ge et al. 2020; Gao et al. 2020). Therefore, it is imperative to determine the regulatory causality of HCC and identify new therapeutic targets.

Autophagy is an evolutionarily conserved degradation pathway through which damaged organelles or misfolded proteins are engulfed into a double-membrane structure and transported to the lysosome for degradation. The contents of autophagosomes are degraded into small molecules (such as nucleotides, amino acids and fatty acids) and pumped out of lysosomes to supply the cell (Yang and Klionsky 2010; Klionsky et al. 2016). Therefore, autophagy is an important inherent response by which cells cope with stress conditions, such as starvation, hypoxia and DNA damage. Autophagy is closely related with many other physiological and pathological processes, including cancer. The process of autophagy is strictly controlled by a series of genes called autophagy-related (ATG) genes to maintain cellular homeostasis (Levine and Kroemer 2008; Shintani and Klionsky 2004).

The p53 gene (TP53) is an important tumor suppressor genes, which plays a broad and powerful function in a variety of cancers, effectively preventing the onset and progression of cancer by responding to various intracellular stressors, such as DNA damage, activating and then promoting DNA repair or guiding the controlled death of aberrant cells, and is known as the “guardian of the genome”. However, p53 gene mutations are prevalent in many cancers and have become an important driver of cancer progression, treatment resistance and poor prognosis. It is important to study the role of p53 gene in tumorigenesis mechanism (Liu and Gu 2022).

In addition to the autophagy-lysosome pathway, the ubiquitin–proteasome system (UPS) is a degradative system in cells. Ubiquitin was identified as the common degron adopted by both degradative systems. Ubiquitin is a small protein containing seven lysine sites, so targeted protein can undergo at least seven kinds of polyubiquitination. K11- and K48-linked polyubiquitination mediates the degradation of proteins through the proteasome, while K63-linked polyubiquitination targets substrates for autophagy degradation and participates in signal transduction and protein translocation (Chau et al. 1989).

The process of ubiquitination involves a cascade of three enzymes, the third of which is an E3 ligase that transfers the ubiquitin from an E2 ubiquitin conjugating enzyme to specific substrates. Thus, it is the E3 ubiquitin ligases that’s the most important one of these three enzymes as it determines substrate specificity and variety (Hershko and Ciechanover 1998). Due to the specificity of E3 ligases, UPS inhibition could be a good strategy for targeted cancer therapy (Hoeller and Dikic 2009; Richardson et al. 2003).

MARCH5/MITOL is one of 11 members of the MARCH family of membrane-bound E3 ubiquitin ligases. MARCH family proteins are localized to the plasma membrane and to the membranes of intracellular organelles such as endosomes, the endoplasmic reticulum (ER), and mitochondria (Bauer et al. 2017). MARCH5 is localized to the outer membrane of the mitochondrion, where it plays an important role in the maintenance of mitochondrial homeostasis. MARCH5 regulates mitochondrial dynamics by ubiquitinating mitochondrial proteins such as Drp1, Fis1, and Mfn1 (Park et al. 2014; Nagashima et al. 2014; Karbowski et al. 2007).MARCH5 is involved in protein quality control and specifically recognizes and binds to mutant superoxide dismutase-1 (SOD-1) and enlarged polyglutamine aggregates accumulating in mitochondria (Lim et al. 2010; Yonashiro et al. 2009; Sugiura et al. 2011). In addition, MARCH5 has been reported to promote autophagy in ovarian cancer and MARCH5 function as a ubiquitination ligase has been reported in several articles. However, the role of MARCH5 in liver tumors has not been clarified, and its related pathways have not been defined.

In this study, we found that MARCH5 promoted autophagy in HCC model, which in turn led to proliferation and migration of HCC cells, and we further found that MARCH5 promoted autophagy through ubiquitination of p53. In animal experiments, we found that overexpression of MARCH5 promoted tumor growth and metastasis by establishing a xenograft model. Taken together, our data suggest that MARCH5-mediated regulation of autophagy mechanism is associated with malignant progression of HCC tumors. These findings may contribute to the development of alternative therapies for HCC patients.

## Material and methods

### Human HCC tissue specimens

Between June 2019 and August 2020, 30 pairs of human HCC tissues and paired normal liver tissues were randomly collected from HCC patients undergoing histopathological examination who were not treated with radiotherapy or chemotherapy before resection. All patients underwent surgery at the First Affiliated Hospital of Zhengzhou University. The study protocol was approved by the Ethics Committee of the First Affiliated Hospital of Zhengzhou University.

#### Cell lines and cell culture

Human hepatocellular carcinoma cell lines (Hep3B, LM3, Huh7, HepG2, MHCC97H) were available from Henan Organ Transplantation Research Centre. Cells were cultured in DMEM (Gibco, USA) supplemented with 1% antibiotics and 10% FBS (Gibco, USA). The incubator was sterilized at 37 °C and 5% CO2.

### Quantitative Real-Time PCR Assay (QRT-PCR)

The overall RNA of HCC cells or clinical specimens was extracted via Trizol (Invitrogen, United States) and then was reversed to cDNA via HiScript® III first Strand cDNA Synthetic Kit (Vazyme, China). The 2 × SYBR Green qPCR Master Mix (Bimake, United States) was utilized for the qRT-PCR assay. Relative quantification of mRNA levels was computed as fold change by the standard formula, 2-△△CT = (CTtarget − CTGAPDH) specimen − (CTtarget − CTGAPDH) control. The sequence of the primers for the QRT-PCR are presented below:MARCH5 sense: 5′- GTCCAGTGGTTTACGTCTTGG-3’; and antisense: 5′- CCGACCATTATTCCTGCTGC-3;

18S sense: 5′- CGCTTCCTTACCTGGTTGAT-3’; and antisense: 5′- GAGCGACCAAAGGAACGATA-3;

### Nuclear protein extraction

The nuclear and cytoplasmic proteins of cells were extracted by the Nucleus and Cytoplasm Abstraction Kit (Solarbio, China) following the supplier’s manual. The entire specimens were reserved at − 80 °C for the assays later on.

### Western blot assay

Cells or tissues were lysed by RIPA buffer added with 1 mM PMSF (Solarbio, China) and Protease Suppressor Cocktail (Thermo Fisher Scientific, United States) and cultivated on ices for 0.5 h. The protein concentrations were identified via the BCA protein analysis kit (Solarbio, China). The specimens were subjected to separation by 10% SDS-PAGE and moved to a PVDF film. Then, the treated films were subjected to 5% nonfat milk in TBST for 120 min under RT and immunoblotted with the specific primary antibody under 4 °C nightlong. The films were subsequently cleaned with TBST and cultivated with alkaline phosphatase-conjugated second antibodies for 60 min under RT. Immunoblotting was observed via the Odyssey® Dlx Imaging System. ImageJ software was utilized to analyze the densitometric.

### Generation of stable MARCH5 overexpressing cell lines

MARCH5 was overexpressed with pLenti-CMV-MARCH5-GFP-Puro plasmid (MARCH5). plenti-CMV-GFP-Puro plasmid was used as a control (Vector). The plasmids were purchased from the Public Protein/Plasmid Library (China), and the whole constructs were verified by sequencing. Briefly, MHCC97H cells were placed in 6-well plates overnight and then transfected with pcDNA3.1/MARCH5 plasmid and pcDNA3.1/Vector plasmid using Lip8000 reagent (SUDGEN, China) according to the supplier's instructions. Twenty-four hours after transfection, cells were selected with 1 μg/ml puromycin (Invitrogen, USA) for 14 days. Stable colonies were screened and stored in 1 μg/ml puromycin.

### Generation of stable MARCH5 knockdown cell lines

MARCH5 was knocked down with pPLK-CMV-MARCH5-sh1-GFP-Puro plasmid (MARCH5). pPLK-CMV-GFP-Puro plasmid was used as control (NC). The plasmids were purchased from the Public Protein/Plasmid Library (China), and the whole constructs were verified by sequencing. Briefly, Huh7 cells were placed in 6-well plates overnight and then transfected with pcDNA3.1/MARCH5-sh1 and pcDNA3.1/MARCH5-sh2 plasmids and pcDNA3.1/NC plasmid using LipoMax reagent (SUDGEN, China) according to the supplier’s instructions. Twenty-four hours after transfection, cells were selected with 1 μg/ml puromycin (Invitrogen, USA) for 14 days. Stable colonies were screened and stored in 1 μg/ml puromycin.

### Cell proliferation analysis

Transfected MHCC97H and Huh7 cells were inoculated into 96-well plates at 1 × 103 per well and assayed on days 1, 2, 3, 4 and 5 post-transfection. Cell activity was assessed by Cell Counting Kit-8 (Solarbio, China). At predetermined time points, the original intermediates in each well were replaced with intermediates containing 10% CCK-8 reagent. After incubation at 37 °C for 120 min, the absorption of the samples was characterised by a microplate reader at 450 nm.

### Colony formation analysis

Transfected MHCC97H and Huh7 cells are grown in 6-well plates (1000 cells per well) and cultured for approximately 14 days. The medium was changed every 3–4 days. The cells were then fixed with 4% PFA and stained with 0.1% CVSS for 15 min. After rinsing with PBS, colony formation was photographed and counted.

### EdU labeling assay

MARCH5 overexpressing MHCC-97H and Huh7 cells after knockdown of MARCH5 were detected using the Click-iT™ EdU Cell Proliferation Kit for Imaging, Alexa Fluor™ 555 dye (C10338, Invitrogen), according to protocols provided by the manufacturer.

### Wound healing assay

Transfected MHCC97H and Huh7 cells were cultured into 6-well plates. When the cells reach 90% fusion, a linear wound is formed with a 200 μL standard pipette after 2 washes with PBS. Cells were incubated in FBS-free intermediate medium for an additional 48 h.

### Cell migration and invasion assay

Transfected MHCC97H and Huh7 cells were inoculated into the upper chamber of Corning cell culture inserts with polycarbonate filter membrane (pore size 8 μm, diameter 6.5 mm). For cell migration capacity, 2 × 105 cells were inoculated with 100 μL of FBS-free intermediate solution into the upper chamber without Matrigel (BD, USA). For cell invasion ability, 2 × 105 cells were inoculated into the upper chamber pre-coated with Matrigel by adding 2 × 105 cells in 100 μL of FBS-free intermediate solution. The lower chamber was used with 800 μL of DMEM intermediate medium (with 20% FBS added). After 48 h of incubation at 37 °C in a humidified environment with 5% carbon dioxide, non-migrated or non-infected cells adhering to the upper chamber were carefully removed, while migrated or infected cells adhering to the lower chamber were treated with 4% PFA for 15 min and stained with 0.1% CVSS for 15 min.

### Xenograft experiment

Animal experiments were approved by the Ethics Committee of the First Affiliated Hospital of Zhengzhou University.5-week-old female BALB/C nude mice were purchased from Beijing Vitality River Laboratory Animal Technology Co Ltd (China) for in vivo proliferative capacity assessment. 5 × 106 MHCC97H cells stably overexpressing MARCH5 were suspended in 100 μL PBS and injected subcutaneously into the right dorsal side of the animals. The cancer volume and body weight of the animals were identified every other day and the average cancer volume was calculated using the formula: volume = (length × width2)/2. After 3 weeks, all the animals were euthanised and imaged, and the cancerous tissues were peeled off and meticulously measured.

### Lung metastasis model

To assess tumor metastasis in vivo, 3 × 106 MHCC97H cells stably overexpressing MARCH5 were suspended in 100 μL of PBS and injected into the animals via the tail vein, and the whole animals were euthanised after 8 weeks. Lung tissues were carefully dissected and photographed. The lungs were stained with H&E. Whole lung tissues of all mice were sectioned and lung metastatic nodules in HPF were counted using microscopic equipment.

### Histological and IHC assays

Clinical samples and lung tissues from animals were RT-fixed in 4.0% PFA night growth solution. Subsequently, the tissues were fixed in paraffin and sectioned (5 μm). Afterwards, lung tissue sections were stained with H&E and observed by light microscopy. For IHC analysis, after deparaffinisation and hydration, the sections were blocked with 3% H_2_O_2_ to block endogenous peroxidase and pre-treated with microwave heating in EDTA (pH 8.0) for 300 s. Sections were stained with DAB and counterstained with hematoxylin. The level of immunostaining was scored separately by two researchers who were unaware of the histopathological features and patient information. The score was determined by a combination of the number of positively stained tumour cells and the intensity of staining.

### Statistical analysis

Data are expressed as mean ± SD of three independent tests. Differences between experimental and control groups were compared by Student’s t-test or one-way ANOVA. Survival rates were calculated by the K-M method, and differences in survival rates were assessed by the log-rank test. Differences were significant at *p < 0.05, **p < 0.01, ***, p < 0.001 when compared to the vector or NC groups. Results were analysed using GraphPad Prism 8.0.

## Results

### MARCH5 is upregulated in HCC tissues and correlates with poor patient prognosis

**Fig. 1:**
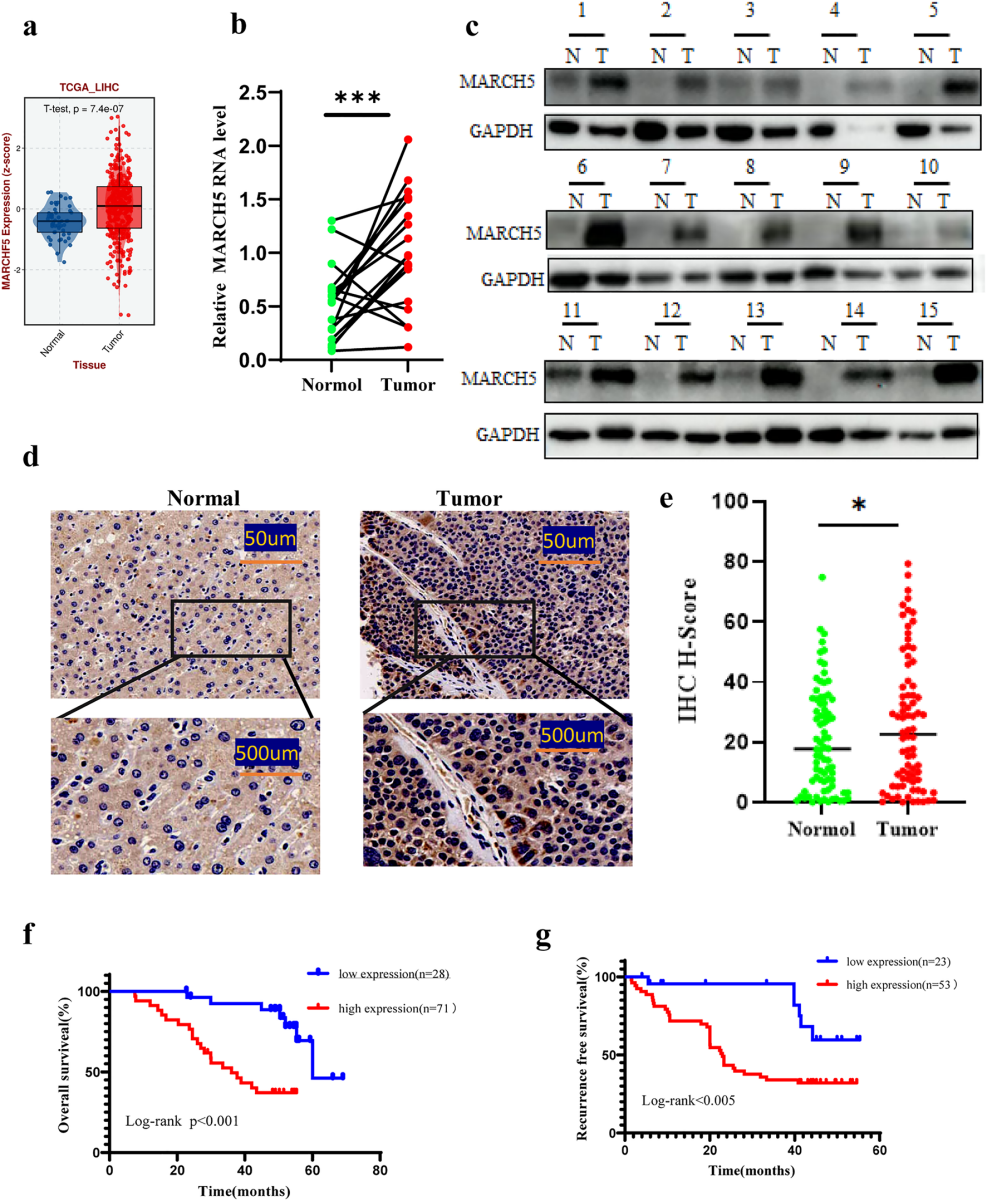
MARCH5 is up-regulated in HCC tissues and correlates with poor prognostic prognosis in patients. **a** Calculated from the TCGA dataset (374 tumor samples and 50 normal samples), the mRNA level of MARCH5 was significantly lower in cancer samples than in normal samples (*P* < 0.001, Student’s t-test). **b** The mRNA level of MARCH5 was detected by QRT-PCR in 24 primary HCC samples and their adjacent healthy tissues. mRNA expression of MARCH5 was higher in HCC tissues than in non-tumor tissues (***p < 0.001, Student's t-test). **c**, **d** The expression of MARCH5 protein in T and NT tissues was detected by immunohistochemistry and Western blotting (n = 15, scale bar: upper 250 μm, lower 100 μm). **e**, **f** According to the immunohistochemical results and divided into two groups, including low expression group and high expression group. Postoperative survival time and recurrence time of HCC patients in the low and high MARCH5 groups were compared by Kaplan–Meier survival analysis (*P* < 0.05)

To determine the level of MARCH5 in HCC, we first examined the mRNA expression of MARCH5 in HCC using the TCGA database. Data analysis of this database showed that MARCH5 was significantly upregulated in HCC tissues compared to normal liver tissues (Fig. 1a). In addition, to verify the expression of MARCH5 in HCC, we extracted total mRNA from 24 HCC tissues and paired adjacent normal liver tissues for QRT-PCR analysis and found that the expression of MARCH5 was significantly increased in HCC tissues (Fig. 1b). Furthermore, we detected MARCH5 in 15 HCC tissues and corresponding adjacent liver tissues by Western blot, which was consistent with the results of QRT-PCR and IHC (Fig. 1c). Additionally, we found by immunohistochemical experiments that the positivity rate of MARCH5 was higher in most cancer tissues than in paracancerous tissues (Fig. 1d, e). Moreover, we were interested in whether MARCH5 affected the survival rate of patients with HCC. In our cohort, patients were divided into low‐ and high‐MARCH5 expression groups. Survival analyses were performed using Kaplan–Meier analysis. Analysis revealed that HCC patients with higher MARCH5 expression exhibited a trend toward poor prognoses, including reduced overall survival and recurrence‐free survival (Fig. 4a, b). These clinical data suggested that MARCH5 plays a critical role in the progression and metastasis of HCC.

### MARCH5 promotes the proliferation of HCC cells in vitro

**Fig. 2:**
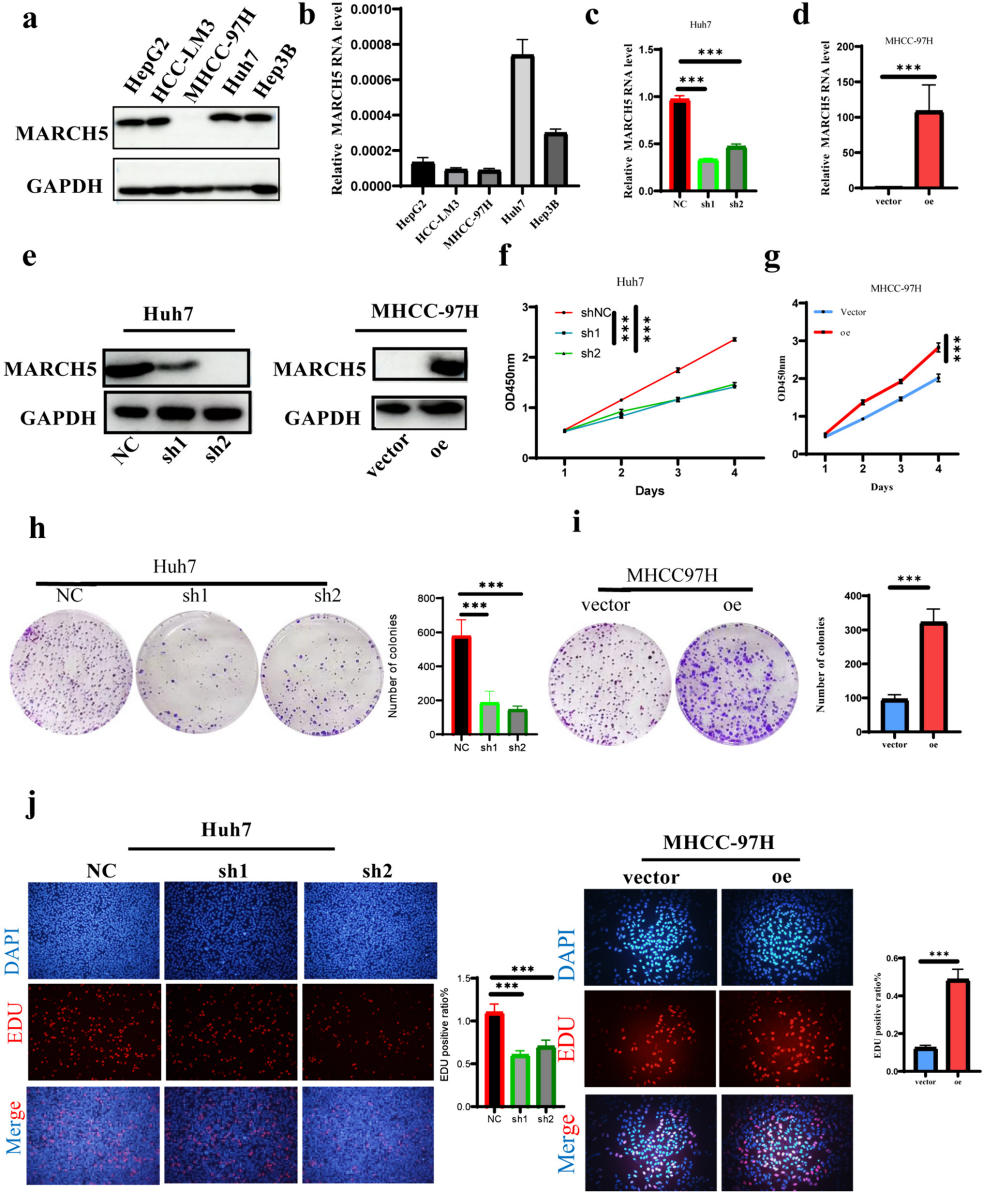
MARCH5 promotes the proliferation of HCC cells in vitro. **a**, **b** The mRNA and protein levels of MARCH5 in human HCC cell lines (Hep3B, MHCC-LM3, Huh7, HepG2, MHCC97H) were detected by QRT-PCR and Western blot. **c**, **e** Successful overexpression of MARCH5 and knockdown of MARCH5 in MHCC97H and Huh7 cells were verified by Western blotting and QRT-PCR. GAPDH was used as an internal loading control. **f**, **g** Cell proliferation capacity was determined by CCK-8 analysis after overexpression or knockdown of MARCH5 in MHCC97H and Huh7 cells. **h**, **i** Effect of MARCH5 on colony formation in HCC cells. **j**, **k** Cell proliferation capacity was determined by EDU analysis after overexpression or knockdown of MARCH5 in MHCC97H and Huh7 cells. *P < 0.05, **P < 0.01, ***P < 0.001, ns no significance

**Fig. 3:**
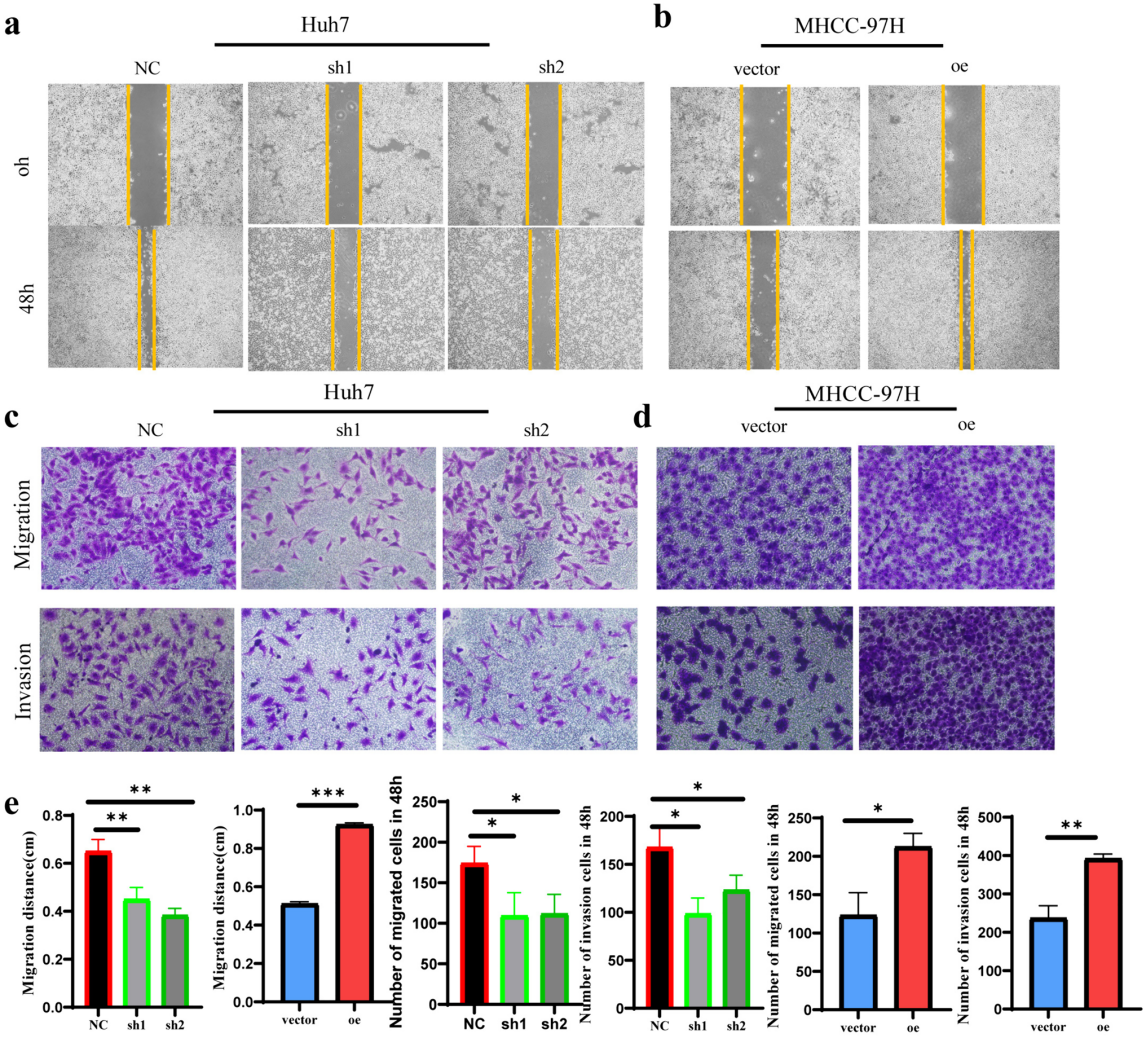
MARCH5 promotes HCC cell migration and invasion in vitro. **a**, **b** The migratory activity of HCC cells after enhancing or knocking down MARCH5 expression was detected by wound healing assay. The wounds were photographed at 0 and 48 h (scale bar, 200 μm), respectively. **c**, **d** Transwell assay showing the migration and invasion of HCC cells after MARCH5 overexpression or MARCH5 deletion (scale bar: 50 μm). *P < 0.05, **P < 0.01, ***, P < 0.001,****P < 0.0001 vs. Vector group or NC group

To investigate the potential function of MARCH5 in HCC, we first examined the expression of MARCH5 in different human HCC cell lines (Hep3B, MHCC-LM3, Huh7, HepG2, and MHCC97H). QRT-PCR and Western blot analyses showed that the expression of MARCH5 varied in each of the five HCC cell lines, but the expression in MHCC-97H was the lowest and highest expression in Huh7 (Fig. 2a, b). Subsequently, we overexpressed MARCH5 in MHCC-97H using plasmids and knocked down MARCH5 in Huh7 cells by two independent small interfering RNAs (siRNAs) and corresponding results were confirmed by both QRT-PCR and Western blot analyses (Fig. 2c, e).By CCK-8 experiments, we could clearly observe that knockdown of MARCH5 significantly inhibited cell proliferation, while overexpression of MARCH5 significantly promoted tumor cell proliferation (Fig. 2f, g). By colony formation assay, smaller colonies appeared in Huh7 cells after knockdown expression of MARCH5 (Fig. 2h). In contrast, overexpression of MARCH5 in MHCC-97H significantly promoted hepatocellular carcinoma cell clone formation (Fig. 3i). To further validate the proliferation-promoting ability of MARCH5, we obtained similar results to the above experiments by EDU experiments i.e. overexpression of MARCH5 then significantly promotes the proliferation of tumor cells and vice versa (Fig. 2j, k). Therefore, these results suggest that MARCH5 promotes the proliferative ability of HCC cells in vitro.

### MARCH5 promotes HCC cell migration and invasion in vitro

To further validate the role of MARCH5 in HCC, we used the wound healing assay to assess the effect of MARCH5 on migration ability of HCC cells. Cells with elevated MARCH5 showed a more pronounced area of wound closure compared to controls, whereas cells with knocked-down MARCH5 presented a narrower area of wound closure (Fig. 3a, b). In addition, the migratory capacity and invasive capacity of MHCC97H and Huh7 cells were investigated using Transwell chambers coated or uncoated with matrigel. Compared with the control group, the migratory and invasive abilities of cells overexpressing MARCH5 were significantly enhanced, whereas the migratory and invasive abilities of cells inhibiting MARCH5 were significantly attenuated (Fig. 3c, d). In order to view the experimental results more intuitively, we statistically analysed the results obtained from each experiment (Fig. 3e). Taken together, the above results strongly demonstrate that overexpression of MARCH5 promotes migration and invasion of HCC cells in vitro.

### MARCH5 promotes growth and metastasis of HCC cells in vivo

**Fig. 4:**
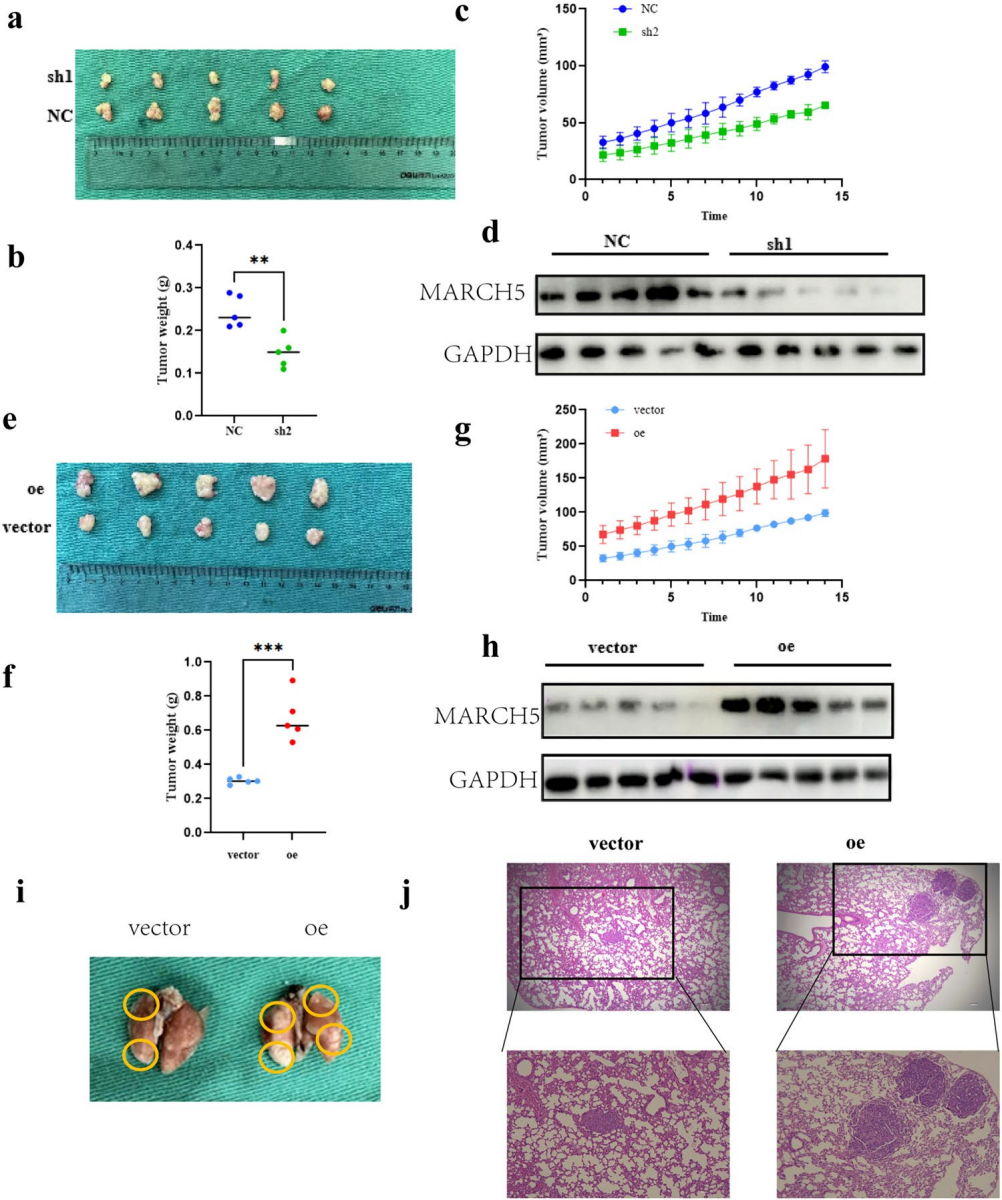
MARCH5 promotes the proliferation and metastasis of HCC cells in vivo. **a** Comparison of tumor growth curves between MARCH5 overexpressing cells and vector control cells. Stable transfected cells of MHCC97H with stable overexpression of MARCH5 and MHCC97H cells with stable knockdown of MARCH5 and control vector stable transfected cells were subcutaneously injected into the right abdominal region of the animals, respectively (5 × 106 cells per mouse, n = 5 per group). Images of BALB/c nude mice in each group 21 days after subcutaneous injection of MARCH5-expressing or MHCC97H cell vectors (**a**, **e**). Weight of tumor nodules in the subcutaneous mouse xenograft model. **b**, **f** Cancer volume was calculated by the formula: volume = (length × width2)/2. **d**, **h** Tumor extracted proteins were verified by WB (**c**, **g**) Number of lung metastases in mice after tail vein injection of MARCH5 overexpressing MHCC97H cells and control cells (3 × 106 cells per mouse, n = 6 per group). **f**, **g** Typical lung and H&E images of lung cancer (scale bar, 250 μm). *P < 0.05, **P < 0.01, ***, P < 0.001

After confirming the effects of MARCH5 on proliferation, migration and invasion in vitro, we investigated the role of MARCH5 in cancer formation in vivo by using a xenograft mouse model. We subcutaneously injected MHCC97H cells stably overexpressing MARCH5 and Huh7 cells knocking down MARCH5 into the bilateral abdomen of mice. Tumors were excised and photographed 3 weeks after inoculation of HCC cells (Fig. 4a, e). Tumor weight was significantly increased in the MARCH5 group compared to the vector group, whereas the tumors of MARCH5 knockout animals were lighter (Fig. 4b, f). Cells overexpressing MARCH5 exhibited higher tumor growth rate and significantly increased tumor volume compared to vector cells, while knockdown of MARCH5 significantly reduced the tumor size (Fig. 4d, h). We also verified the inhibitory effect of MARCH5 on distant seeding of HCC cells using the tail vein injection model. Eight weeks after injection, the number of metastatic nodules in mice injected with overexpressed MARCH5 group was significantly more than that in the control group (Fig. 4i, j). These results were consistent with the in vitro findings, indicating that MARCH5 promoted the proliferation and metastasis of HCC cells in vivo.

### MARCH5 promotes malignant progression of hepatocellular carcinoma by regulating autophagy activation

**Fig. 5:**
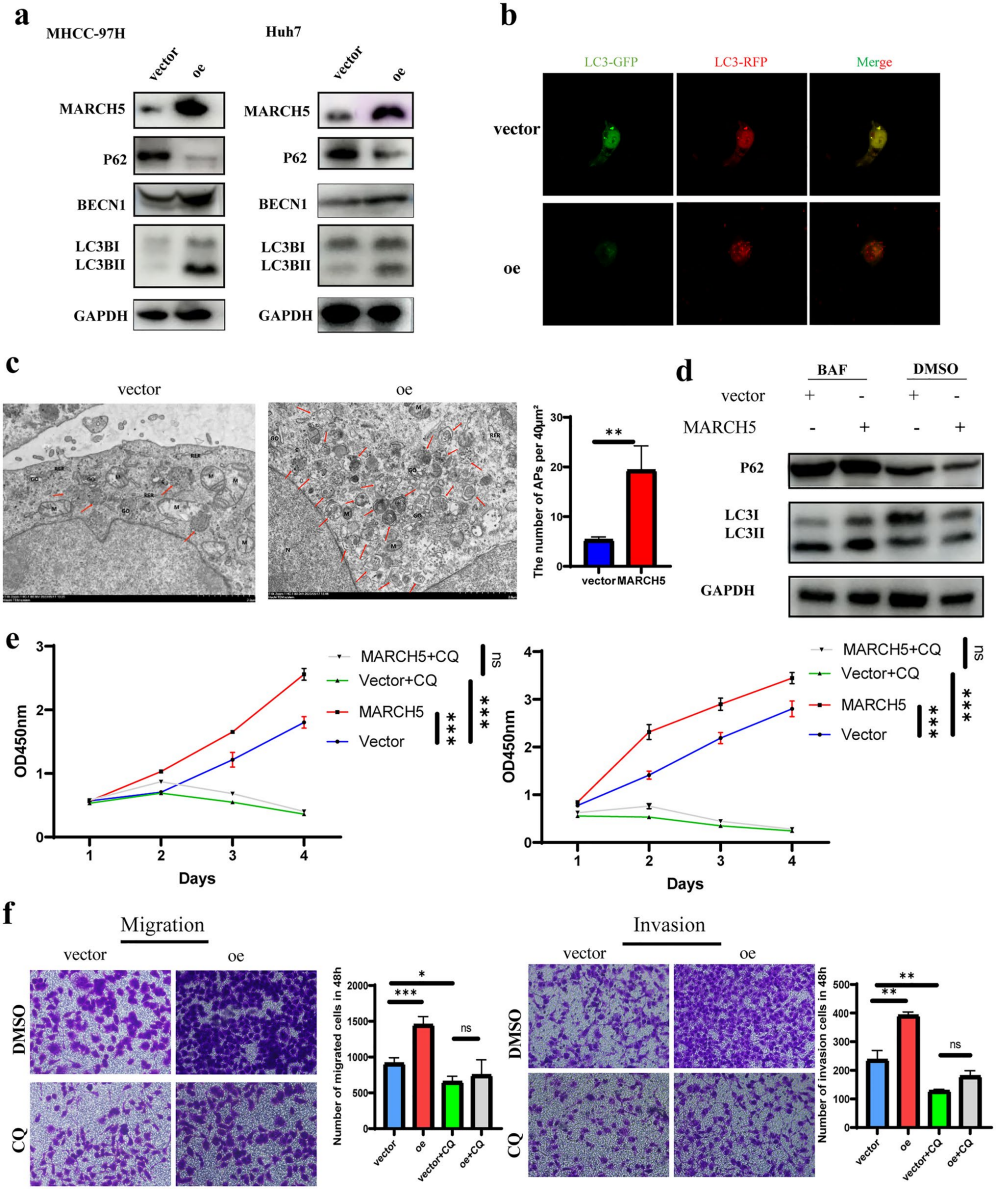
MARCH5 promotes malignant progression of hepatocellular carcinoma by regulating autophagy activation. **a** MHCC-97H and Huh7 cells stably expressing MARCH5 affect the expression of autophagy-related proteins detected by immunoblotting. Data are representative immunoblots of three independent assays. **b** Control or MHCC-97H and Huh7 cells overexpressing MARCH5 were infected with adenovirus with mRFP-GFP-LC3. Autophagic flux was detected by live cell imaging microscopy. Images show red autophagic lysosomes or red/green dual CN_FFFE coloured autophagosomes. Zoom bar = 25 μm. **c** Electron microscopy of stable MHCC-97H and Huh7 cells. Typical autophagy lysosomes observed in MHCC-97H cells overexpressing MARCH5. Autophagic lysosomes are shown by arrows. Zoom bar = 1–2 μm. **d** MHCC-97H cells expressing vector or MARCH5 were treated with or without bafilomycin (400 nM) for 12 h and the expression of p62 and LC3B-II/I was detected by immunoblotting. **e** Proliferation of MHCC97H and Huh7 cells overexpressing MARCH5 under CQ treatment was detected by CCK-8. **f** Transwell assay showing the migration and invasion.of HCC cells overexpressing MARCH5 and CQ treatment (scale bar, 50 μm). CQ: 2 μmol/ml. *P < 0.05, **P < 0.01, ***, P < 0.001, compared *P < 0.05, **P < 0.01, ***P < 0.001, ns no significance

Next, we aimed to elucidate the molecular mechanisms by which MARCH5 promotes malignant progression in hepatocellular carcinoma. Autophagy is necessary to maintain malignant progression in various tumors. MARCH5 has been reported to activate autophagy in other cancers. By GO enrichment, we similarly found the same conclusion (Figure S1a). This prompted us to hypothesize that MARCH5 may exert biological effects on hepatocellular carcinoma through activation of autophagy. Therefore, we investigated the effect of MARCH5 on autophagy in hepatocytes, which is consistent with the malignant biological behavior of HCC, and found that stable overexpression of MARCH5 strongly increased the induced transactivation of BECN1 and microtubule-associated protein 1 light chain 3 (LC3)-II, and decreased the p62 level (Fig. 5a). To verify the role of MARCH5 in the regulation of autophagic flux, the flux rate of autophagy was measured using mRFP-GFP. A large number of red dots (autophagosomes) appeared in Fig. 5b, and more autophagosomes and autolysosomes were found in MARCH5 gene overexpressing cells compared to controls. Transmission electron microscopy showed that overexpression of MARCH5 increased the number of autophagosomes and autolysosomes in MHCC-97H cells (Fig. 5c). The late autophagy inhibitor bafilomycin also increased MARCH5-induced LC3B-II accumulation in Huh7 and MHCC-97H cells (Fig. 5d). Taken together, these data suggest that MARCH5 activates autophagy in hcc. To further validate the role of MARCH5-activated autophagy in hepatocellular carcinoma progression, we first blocked autophagy activation by adding the autophagy inhibitor CQ. The results showed that the proliferation, invasion and migration of tumor cells overexpressing MARCH5 were significantly blocked by the addition of the autophagy inhibitor (Fig. 5e, f). Taken together, these results suggest that MARCH5 promotes the malignant progression of hepatocellular carcinoma by affecting autophagy.

### MARCH5 promotes the malignant progression and autophagy of HCC by ubiquitination degradation of p53

Next, we investigated the molecular mechanisms by which MARCH5 promotes malignancy and autophagy in hepatocellular carcinoma. The tumor-promoting and autophagy-promoting effects of MARCH5 have been reported in several papers. We found that MARCH5 was closely associated with K48-linked ubiquitination, tricarboxylic acid cycle, activation of autophagy, and p53 pathway in hepatocellular carcinoma by GO and KEGG enrichment analysis(Figures S1a, b). Due to the critical role of p53, a cancer inhibitor gene, in autophagy and tumorigenesis, we hypothesized that MARCH5 could regulate malignant progression and autophagy in hepatocellular carcinoma through the p53 signalling pathway.

**Fig. 6:**
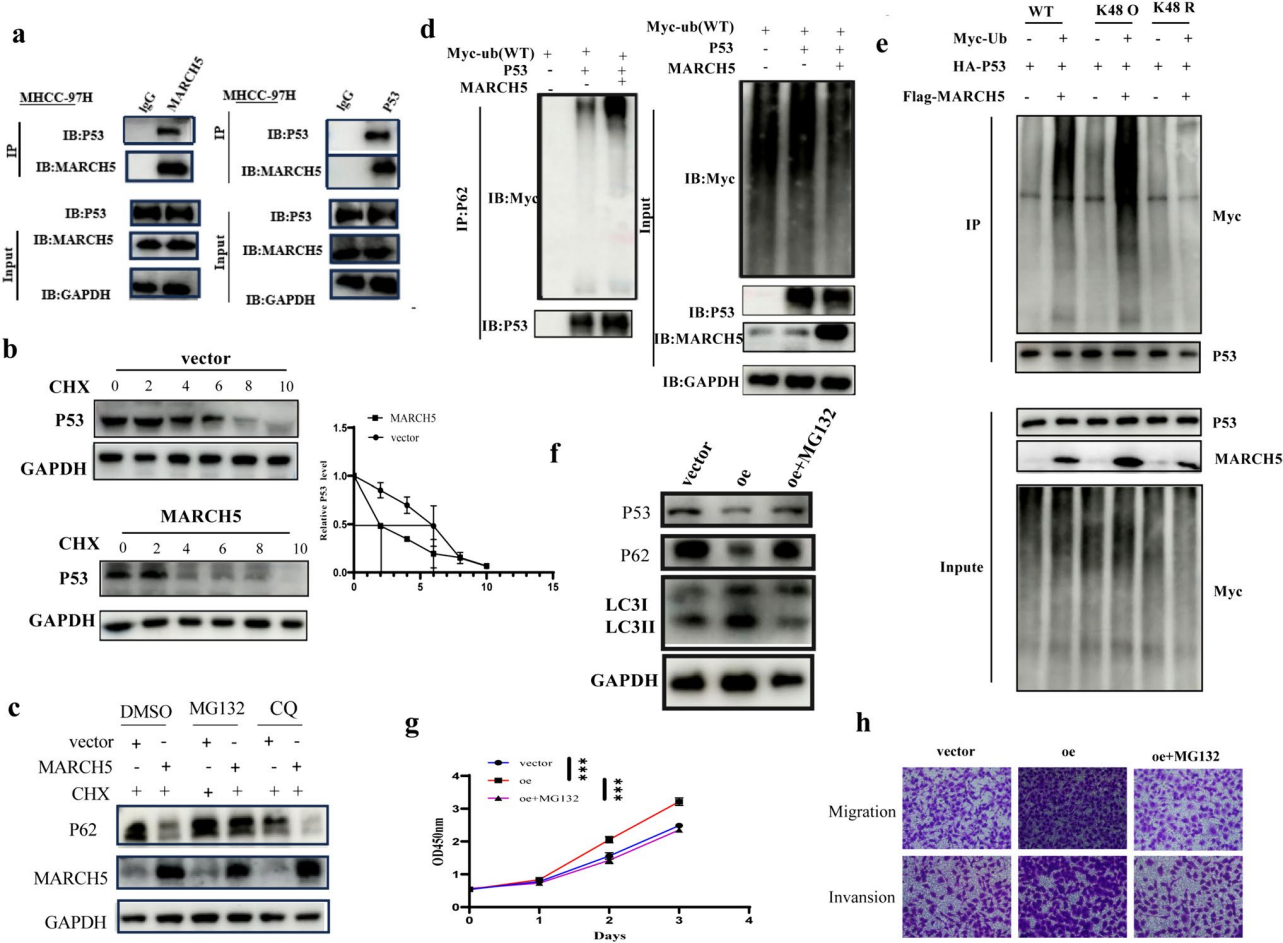
MARCH5 promotes the malignant progression and autophagy of HCC by ubiquitination degradation of p53. **a** MHCC-97H extracts were co-immunoprecipitated (IP) with anti-MARCH5 antibody or IgG followed by anti-p53 antibody (left) and IP with anti-p53 antibody or IgG followed by blotting with anti-MARCH5 antibody (right panel). **b** MARCH5 regulates the degradation of p53 protein. MHCC-97H cells transfected with MARCH5 were treated with 20 μM CHX for the indicated time, and the protein extracts were subjected to the immunoblotting assay. **c** MARCH5 promotes the proteasomal degradation of p53. MHCC-97H transient overexpressing with MARCH5 were treated with 100 nM CQ or 10 μM MG132 for 6 h, and then assayed for immunoblotting.Knockdown of MARCH5 does not affect the transcription of p53. **d** MHCC-97H cells were transfected with transfected ubiquitin-Flag, p53-HA, and pcDNA-MARCH5 for 48 h, and then treated with MG132 (10 μmol/L) for 4 h. The cells were then incubated with cycloheximide (20 μmol/L) until the indicated time points. Cell lysates were immunoprecipitated using anti-HA antibody. Data are representative immunoblots from three independent experiments. **e** 293 T cells were transfected with different types of ubiquitin plasmids and screened for ubiquitin sites of MARCH5 action on P53 by Western Blot. **f** Stable overexpression of MARCH5 compared to the vector group and as well as overexpression of MARCH5 after MG132 treatment affects the expression of autophagy-related proteins detected by immunoblotting. Data are representative immunoblots from three independent experiments. **g**, **h** Stable overexpression of MARCH5 and overexpression of MARCH5 after MG132 treatment were verified to affect the proliferation, invasion and migration of tumor cells compared with the vector group by CCK8 and transwell assays

To further explore the regulatory role of MARCH5 on p53 protein, we confirmed the interaction between MARCH5 and p53 by immunoprecipitation (Co-IP) results (Fig. 6a). We examined the expression of MARCH5 and p53. We designed siRNA to knock down MARCH5 and plasmids to overexpress MARCH5. Knockdown of MARCH5 with siRNA in liver cancer cells increased p53 protein levels, while overexpression of MARCH5 decreased p53 protein levels (Fig. S1c). However, when MARCH5 was knocked down, there was no significant change in p53 mRNA expression (Figure S1d). Because MARCH5 acts as a member of the E3 ubiquitin ligases, we hypothesized that MARCH5 mediates the degradation of p53 rather than altering its transcription. Cycloheximide (CHX) non-selectively blocks protein biosynthesis and is therefore widely used to measure the half-life of specific proteins in cells. After treating hepatocellular carcinoma cells with CHX, we found that overexpression of MARCH5 significantly shortened the half-life of the p53 protein, further validating our hypothesis (Fig. 6b). The proteasome inhibitor MG132 rescued the p53 degradation mediated by MARCH5 overexpression. This further confirmed that MARCH5 can degrade p53 (Fig. 6c). Further experiments showed that overexpression of MARCH5 significantly promoted total ubiquitination of p53 (Fig. 6d); by transfecting ubiquitin molecules plasmids with different site activities, we found that MARCH5 promoted K48-linked polyubiquitination-mediated degradation of p53 (Fig. 6e). The above results indicate that P53, as a ubiquitination substrate of MARCH5, can be ubiquitinated by MARCH5, and this ubiquitination is mediated in a K48-linked manner. Finally, the ubiquitination inhibitor MG132 reversed MARCH5 ubiquitination degradation of P53, elevated P53 levels and increased levels of the autophagy-related marker P62 (Fig. 6g, h), and markedly inhibited the pro-oncogenic effects of MARCH5 (Fig. 6g, h). Collectively, these data suggest that MARCH5 promotes malignant progression and autophagy in hepatocellular carcinoma by regulating ubiquitination of p53.

## Discussion

Previously, there have been numerous reports on the pro-carcinogenic role of MARCH5 in breast, melanoma, colorectal and lymphoma as well as drug resistance and promotion of mitochondrial autophagy.To date, the extremely mechanism of the effect of MARCH5 on the development of HCC has not been clarified. Based on the results derived from the TCGA database, clinical samples, we found that MARCH5 was significantly upregulated in human HCC samples compared to neighbouring normal tissues. In addition, patients with high MARCH5 expression had shorter OS compared to patients with low NARCH5 expression. These findings suggest that MARCH5 may play a promoting role in HCC development. To explore the speculation, we stably overexpressed MARCH5 in MHCC97H cells, and the results showed that overexpression of MARCH5 in vitro significantly promoted cell proliferation, migration and invasion ability. whereas silencing of MARCH5 in Huh7 cells led to the opposite effect. We also demonstrated the promotional effect of MARCH5 on the growth and metastasis of hepatocellular carcinoma cells in a nude mouse rat model. Thus, our findings reveal that MARCH5 promotes the malignant progression of hepatocellular carcinoma.

The pathogenesis of HCC has been extensively studied, and there is growing evidence that autophagy is involved in the malignant development of tumors. Impaired autophagy leads to multiple pathological conditions in humans, including liver dysfunction and tumorigenesis (39). Conversely, autophagy provides nutrients to tumor cells during tumor progression and promotes invasive migration and drug resistance by facilitating EMT. For example, recent studies have shown punctate staining or increased expression of microtubule-associated protein 1 light chain 3 beta (LC3B) in various metastatic tumors, suggesting that autophagy is upregulated during metastasis (Peng et al. 2013; Lazova et al. 2012; Zhao et al. 2013; Han et al. 2011). Metastatic cancer cells have been reported to increase ATP synthesis to support their motility by enhancing mitochondrial activity and promoting catabolism, including autophagy (Benzarti et al. 2020; Thirupathi and Chang 2019). In hepatocellular carcinoma, autophagy is required for transforming growth factor-β (TFG-β)-induced EMT (Li et al. 2013), as well as for the production of pro-invasive cytokines such as interleukin-6 (IL-6), matrix metalloproteinase-2 (MMP-2), and Wnt family member 5A (WNT5A) during invasion of the RAS-transformed cancer cell line (Lock et al. 2014). Recently, Yamamoto et al. (Yamamoto et al. 2020) demonstrated that autophagy degrades major histocompatibility complex class I (MHC-I) in pancreatic cancer, thereby promoting immune evasion. In the present study, we provide evidence that high MARCH5 accompanied by increased autophagy is associated with malignant progression of liver tumors and low survival in HCC patients and reveal that MARCH5 promotes autophagy through ubiquitinated p53 leading to tumor cell proliferation.Thus, our mechanistic studies and clinical data strongly suggest that MARCH5 is an important player in hepatocarcinogenesis.

The major Antioncogene p53 accumulates in cells in response to DNA damage, oncogene activation, and other stresses (Donehower et al. 2019; Morselli et al. 2011). We found that MARCH5 is closely associated with autophagy, the p53 signalling pathway, and ubiquitination in hepatocellular carcinoma by bioinformatics prediction. Considering the critical role of p53, an oncogene, in autophagy and tumourigenesis and the promotion of MARCH5 as an E3 ubiquitin ligase for autophagy. Therefore, we speculate that MARCH5 may regulate autophagy in hepatocellular carcinoma through the p53 signalling pathway. Studies have shown that p53 inhibits autophagy by interacting with FIP200, which is homologous to yeast ATG17 (Morselli et al. 2011). Knockdown of p53 in CRC promotes the conversion of LC3I to LC3II and the expression of Beclin-1, leading to the activation of autophagy (Livesey et al. 2012). By evaluating the changes in the mRNA and protein levels of p53 after MARCH5 stimulation, we demonstrated that MARCH5 affects posttranslational modifications of p53 proteins. The ubiquitin–proteasome system (UPS) is the most important mechanism for p53 degradation (Allende-Vega and Saville 2010). IL-13/IL-13RA2 signalling promotes stemness, proliferation and invasive migration in colorectal cancer by inducing p53 ubiquitination degradation (He et al. 2024).Therefore, we hypothesized that the autophagy activation induced by MARCH5 might be mediated by p53 ubiquitination. Our results essentially confirm that MARCH5 stimulation promotes p53 ubiquitination. Furthermore, experimental validation confirmed that MARCH5 can interact with p53 and that ubiquitination degrades p53 and activates autophagy. Consistently, the autophagy inhibitor CQ indeed reversed MARCH5 stimulation-induced autophagy activation and oncogenic function. And the ubiquitination inhibitor MG132 also reversed autophagy induced by MARCH5 stimulation, including autophagy-related markers LC3 II/I. ratio and P62 protein level. In conclusion, MARCH5 promotes malignancy and autophagy in hepatocellular carcinoma through ubiquitination and degradation of p53.

In summary, our study reveals for the first time a novel HCC-promoting role of MARCH5 in degrading p53 via ubiquitination, thereby enhancing HCC proliferation and metastasis by promoting autophagy. Targeting MARCH5-mediated p53 degradation may be a potential strategy for HCC prevention and treatment. These findings provide an important basis for understanding HCC proliferation and metastasis and identify MARCH5 as a diagnostic and therapeutic target for HCC patients.

## Conclusion

Our findings suggest that MARCH5 promotes autophagy through ubiquitination degradation of p53, thereby affecting tumour proliferation, invasion and migration. These findings open an avenue for the treatment of HCC.

### Supplementary Information

Below is the link to the electronic supplementary material.

Supplementary file1 (TIF 16559 KB)

## Data Availability

The original contributions to this study are included in the article/supplementary material. For further inquiries, please contact the corresponding author.

## Abbreviations

HCC: Hepatocellular carcinoma
MARCH5: Membrane-associatedRING-CH5
EMT: Epithelial-mesenchymal transition
ATG: Autophagy-related genes
LC3: Microtubule-associated protein light chain 3
GFP: Green fluorescent protein
OS: Overall survival
