# Supplementary figures and images for: MARCH5 promotes hepatocellular carcinoma progression by inducing p53 ubiquitination degradation
Source: J Cancer Res Clin Oncol. 2024 Jun 11;150(6):303. doi: 10.1007/s00432-024-05782-7 (PMC11166841; doi:10.1007/s00432-024-05782-7)

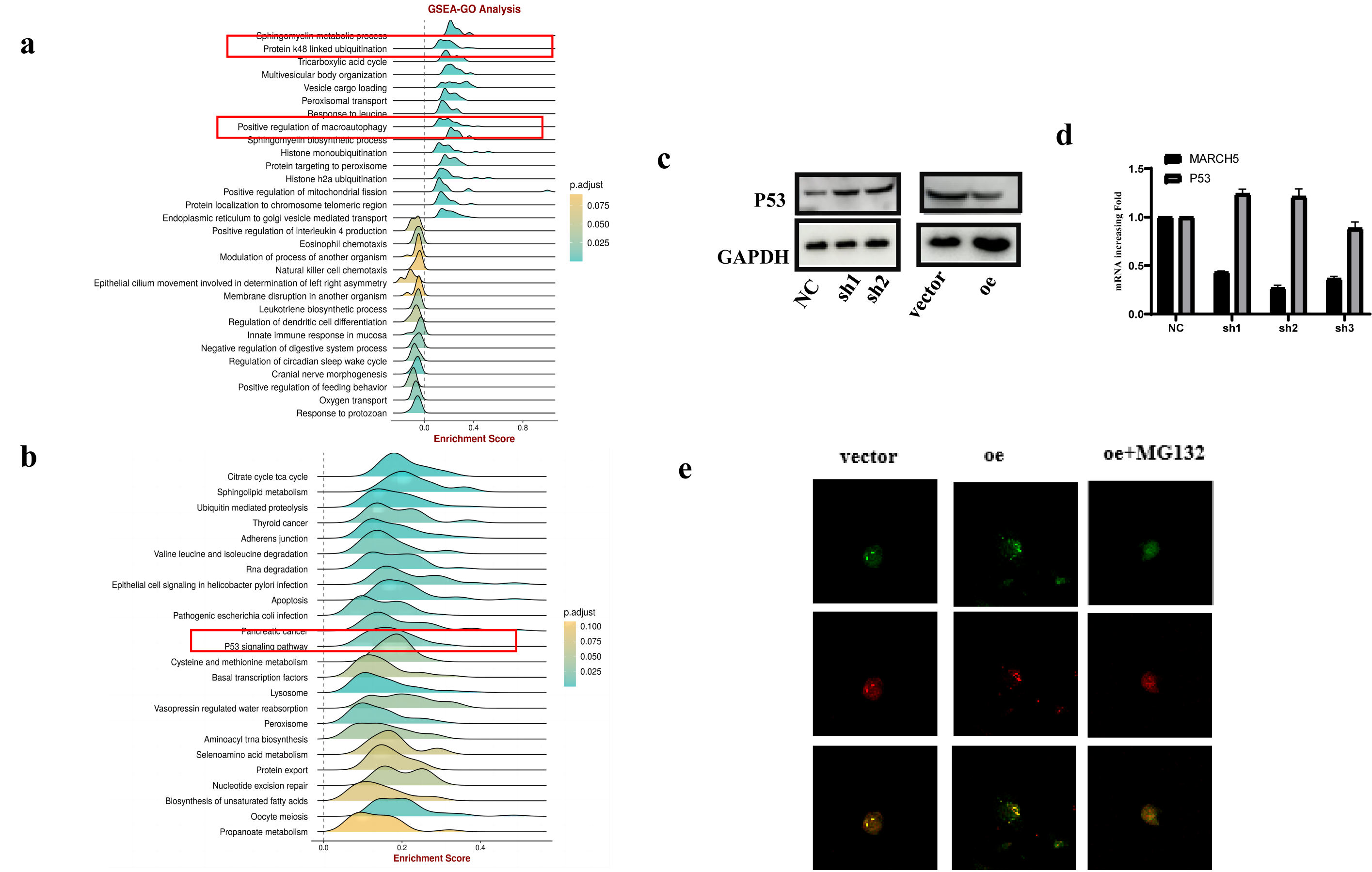

Supplement: Supplementary file 1 — Supplementary file1 (TIF 16559 KB) [file 432_2024_5782_MOESM1_ESM.tif]
